# The use of sleep aids among Emergency Medicine residents: a web based survey

**DOI:** 10.1186/1472-6963-6-136

**Published:** 2006-10-19

**Authors:** Daniel A Handel, Ali Raja, Christopher J Lindsell

**Affiliations:** 1 Department of Emergency Medicine, University of Cincinnati Medical Center, Cincinnati, OH 45267, USA; 2 Now at Center for Policy and Research in Emergency Medicine, Department of Emergency Medicine, Oregon Health and Sciences University, Portland, OR, USA

## Abstract

**Background:**

Sleepiness is a significant problem among residents due to chronic sleep deprivation. Recent studies have highlighted medical errors due to resident sleep deprivation. We hypothesized residents routinely use pharmacologic sleep aids to manage their sleep deprivation and reduce sleepiness.

**Methods:**

A web-based survey of US allopathic Emergency Medicine (EM) residents was conducted during September 2004. All EM residency program directors were asked to invite their residents to participate. E-mail with reminders was used to solicit participation. Direct questions about use of alcohol and medications to facilitate sleep, and questions requesting details of sleep aids were included.

**Results:**

Of 3,971 EM residents, 602 (16%) replied to the survey. Respondents were 71% male, 78% white, and mean (SD) age was 30 (4) years, which is similar to the entire EM resident population reported by the ACGME. There were 32% 1st year, 32% 2nd year, 28% 3rd year, and 8% 4th year residents. The Epworth Sleepiness Scale (ESS) showed 38% of residents were excessively sleepy (ESS 11–16) and 7% were severely sleepy (ESS>16). 46% (95 CI 42%–50%) regularly used alcohol, antihistamines, sleep adjuncts, benzodiazepines, or muscle relaxants to help them fall or stay asleep. Study limitations include low response and self-report.

**Conclusion:**

Even with a low response rate, sleep aid use among EM residents may be common. How this affects performance, well-being, and health remains unknown.

## Background

Sleep deprivation among resident physicians is of growing concern. Sleep deprivation is a cause of fatigue, which is characterized by decreased work capacity and efficiency accompanied by feelings of weariness, sleepiness, or tiredness [1]. Research on resident sleep deprivation has primarily focused on the detrimental impact of fatigue on patient care [1-3]. Various specialties, including Obstetrics and Gynecology [4], General Surgery [5], Pediatrics [6], and Internal Medicine [2,3] have shown sleep deprivation among residents negatively affects performance. However, fatigue also affects resident well-being and quality of life [7,8]. Fatigue arising from extended work shifts and shift work have been implicated as impacting both physical and psychological disorders, ranging from an increased risk of motor vehicle crashes [9,10], depression [11], and heart disease [12], to drug and alcohol abuse [13-15]. Recently, research has begun to focus on strategies for maintaining wakefulness and decreasing fatigue while on shift [16]. To fully counteract the negative effects of fatigue, however, there is an overwhelming need for interventions to minimize fatigue induced by sleep deprivation. In the absence of sweeping changes to the system of resident education, further decreases in work hours, and abandonment of rotating shifts, few solutions remain.

We contend that many residents have recognized this conundrum and have taken steps to reduce their personal sleep deprivation by improving their likelihood of sleep during off duty hours through the use of pharmacological sleep aids. We aimed to determine the prevalence of sleep aid use among Emergency Medicine (EM) residents. We additionally sought to preliminarily explore how their use may relate to perceptions of sleepiness, stressors and work experience.

## Methods

### Study design

We conducted an anonymous web-based survey study of residents at allopathic Emergency Medicine residency programs throughout the United States during 2004. All 3,791 residents enrolled at the time of this survey were eligible. The local Institutional Review Board approved the study.

### Survey instrument

The survey instrument was developed as a component of an educational project. The educational project involved the design of a questionnaire to ascertain the use of sleep aids, local implementation of the survey, and analysis of results. The instrument was developed based on the experiential input from 40 emergency medicine residents participating in the educational program. The stated aim was to solicit information about sleep aid use during the current year of residency.

The Epworth Sleepiness Scale (ESS) was included as the primary measure of sleepiness [17]. The ESS ranges from 0 to 24, and can be used to categorize respondents as normal (ESS = 10), suffering from excessive sleepiness (ESS 11–16), or suffering from severe sleepiness (ESS>16). Two questions about sleep deprivation were asked: i) causes of fatigue during waking hours, and ii) causes of difficulties falling asleep. These domains were expressed as being specific and separable. To obtain response categories, residents pooled their own experiences to derive definable categories that captured the problems they felt caused their fatigue or difficulties in falling asleep.

Information on sleep aid use was asked first by direct questions on methods used to initiate sleep, and then by more detailed questions about over-the-counter, prescription, and non-prescription pharmacological sleep aid use. We elected not to ask questions related to restricted drugs to minimize ethical concerns.

All 40 residents participating in the educational project were asked to complete the draft questionnaire. Response rate was 65%. Data were entered into an electronic format and analyzed interactively during didactic sessions with the participants. Content and construct validity were satisfactory based upon informal evaluation of responses; there were no inconsistencies found and residents indicated that they provided the expected information. In this pilot project, no information was requested about the use of stimulants. However, several respondents volunteered information on stimulant use and so an opportunity to declare stimulants was added to the questionnaire. In addition, participating in moonlighting activities (i.e. ED shifts beyond those expected by the residency program) was identified as a possible source of fatigue not captured in the pilot survey instrument. The final survey instrument is given in the Appendix (See Additional file: 1).

### Survey methodology

The questionnaire was coded into a secure, password-protected web site using ColdFusion (Macromedia Inc, San Francisco, CA), and the data were captured into an Oracle database (Oracle Corporation, Redwood Shores, CA). Completion of a questionnaire resulted in creation of a new record in the database that was simultaneously written to two mirrored hard disk drives. Additionally, data were backed up daily.

Residency directors at all 130 institutions with an Emergency Medicine residency program were solicited to forward the invitation to participate to their residents; our initial attempt to obtain residents' e-mail addresses for direct invitation was resisted by the majority of responding residency directors, citing local privacy policies. The invitation to participate included an introduction describing the overall objectives of the study, statements of anonymity of responses, and an invitation to participate in a drawing for a $100 Amazon.com gift card on completion of the survey. Specific instructions were to log onto the web site using the provided link, to enter the web site only once, and to complete the survey. Participants were told the survey would take between five and ten minutes to complete, and that they should complete the survey within one month.

When a resident used the link in their e-mail to connect to the web site, they were logged onto the survey. On completion of the final survey question, they were asked if they wished to participate in the drawing for the gift certificate. If they indicated a desire to do so, they were requested to provide an e-mail address to which the gift certificate could be sent. Their e-mail identity was not linked to their responses in any way.

After two weeks, reminder e-mails, also containing the link, were e-mailed to the residency directors for distribution. An additional reminder was sent after three weeks. Residency directors were also asked to promote the survey and its importance for understanding resident well being. After six weeks, the web site was closed and data were extracted for analysis. The drawing for the gift certificate was held at this time.

Because of the potential for respondents to provide incriminating responses, we elected to conduct this survey with anonymous responses. Because we collected demographic data, and unlinked e-mail addresses for participation in a drawing for a gift certificate, our survey design included blind deletion of all data if less than ten responses were obtained. If less than ten responses had been obtained, it may have been feasible for us to link a response to an individual.

### Data analysis

Respondents are described using means and standard deviations for continuous variables and frequencies and percents for categorical variables. The primary outcome measure for this study is the proportion of residents using sleep aids, 95% confidence intervals of this proportion have been computed using the score method with continuity correction. Agreement between response categories have been assessed using the Phi statistic where appropriate.

We used univariable and multivariable logistic regression to explore associations between perceptions of fatigue, stressors, work experience, and the use of sleep aids. Multivariable models were created using both forwards and backwards stepwise procedures; comparison of models obtained using both the forwards and the backwards methods can be used to identify problematic colinearity and confounding. This analysis was conducted to explore potential relationships only; we aimed to develop preliminary data that could identify variables that would warrant appropriate operationalization in future studies. Analyses used SPSS v 14.0 (SPSS Inc, Chicago, IL) and Microsoft Excel (Microsoft Corporation, Redmond, WA).

## Results

There were 823/3791 individuals (20.7% of all EM residents) who logged onto the website. Multiple choice questions (question 1 to 8 and demographics) were completed by 602/3791 (15.9%) and detailed questions on sleep aid and stimulant use (questions 9 to 11) were completed by 458/3791 respondents (12.1%).

Among the 602 residents responding to multiple choice questions, mean age was 30 years (SD 4 years), 71.4% were male. Race was 78% white, 11% Asian, 4% black and 4.0% other, race was not given for 3%. Hispanics comprised 4% of respondents. Residency format was PGY1–3 for 71%, PGY1–4 for 14%, and PGY2–4 for 14%. There were 32% 1st year, 32% 2nd year, 28% 3rd year, and 8% 4th year residents. For all EM residents in 2004, the ACGME reported a mean age of 31.2 years, 64% males, with 33% in the first year, 32% in the second year, 31% in the third year, and 4% in the fourth year of residency [18].

**Figure 1:**
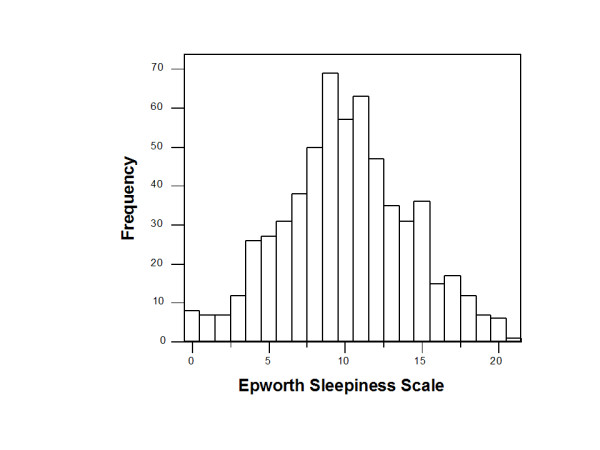
Distribution of the Epworth Sleepiness Scale score.

The mean Epworth Sleepiness Scale (ESS) score was 10.0 (SD 4.2, Figure 1). The ESS was normal (<10) in 332/602 (55%), indicative of excessive sleepiness in 227/602 (38%), and indicative of severe sleepiness in 43/602 (7%) of residents. The time taken to fall asleep was more than 10 minutes for 22%, between 5 and 10 minutes for 40%, and less than 5 minutes for 38% of respondents.

Factors causing fatigue and difficulties initiating sleep are shown in Table 1. To differentiate usual causes of fatigue from extraordinary events, residents were asked to report factors only if present at least four times a month for two consecutive months. In addition to pre-specified factors, residents volunteered that maintaining sociality (n = 8), problems sleeping (n = 5), relationship problems (n = 4), eating habits (n = 4), financial stress (n = 2), medications (n = 2), and hormonal cycles (n = 1) were causes of fatigue. Financial stress (n = 1), stress over household maintenance (n = 2), lack of exercise (n = 1), pregnancy (n = 1), and health problems (n = 1) were identified as causes of difficulty in initiating sleep.

### Sleep aid use

Methods that respondents used to help them fall asleep more than four times a month for at least two months included medication in 17% (102/602), alcohol in 9% (57/602) of cases, and both medications and alcohol in 8% (48/602) of respondents; overall 34.4% (95 CI 30.6% to 38.4%) responded positively when asked directly if they used medication or alcohol to help them fall asleep. Reading (61%), television (42%), sexual activity (36%) and meditation (13%) were also used.

**Figure 2:**
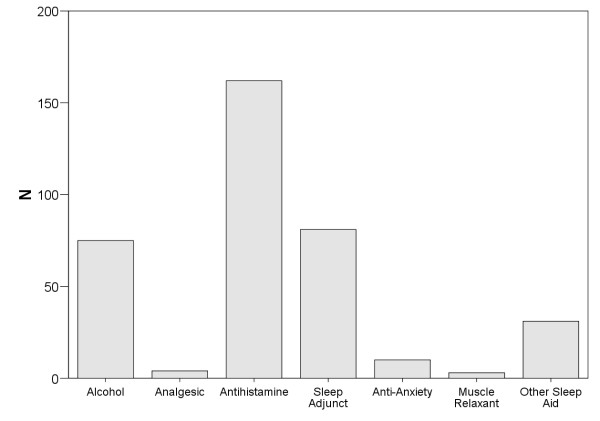
Sleep aids used by the 458 respondents who provided detailed information.

Of the 458 residents responding to detailed question on sleep aid use, 255 (55.7%, 95 CI 51.0% to 60.3%) reported the use of aids to fall or stay asleep, including pharmaceuticals, homeopathic remedies, and alcohol. Types of sleep aid are shown in Figure 2. Antihistamines were primarily diphenhydramine, although two respondents used a doxylamine-based product. Analgesics included oxycodone (n = 2) and acetaminophen with codeine (n = 1). Sleep adjuncts were melatonin supplements, zolpidem, and zaleplon. Anti-anxiety medications were primarily benzodiazepines although one respondent used buspirone and two used citalopram. Sleep aids characterized as 'other' included vitamins, herbal remedies, and anti-emetics.

We compared responses to the direct question on use of medications or alcohol to fall asleep to the detailed reports of sleep aid use (Table 2). Overall, agreement between the two questions was modest (Phi = 0.684). Combining responses to obtain an estimate of the maximum number of residents using a sleep aid suggests 278 of the 602 respondents (46.2%, 95 CI 42.2% to 50.3%) used some form of sleep aid. Exclusive of alcohol, 234 of the 602 respondents (38.9%, 95 CI 35.0% to 42.9%) used a sleep aid.

### Stimulant use

There were 380/458 residents describing stimulant use to help them stay awake (83.0%, 95 CI 79.1–86.2%). The majority of stimulants were caffeine based, both tablet and drink. Amphetamine use was reported by five respondents, methylphenidate hydrochloride by two respondents, modafinil by five respondents, and ephedrine by six respondents. Nicotine gum and tobacco products were used as stimulants by 21 respondents.

### Factors related to sleep aid use

Table 3 shows the results of univariable logistic regression models exploring the influence of year of training, program type, moonlighting, causes of fatigue, causes of difficulty initiating sleep, sleepiness, and demographics on the odds of using sleep aids. In these univariable models, respondents who identified positively with the various causes of fatigue and causes of difficulty initiating sleep were significantly more likely to use sleep aids, both inclusive and exclusive of alcohol.

Table 4 shows the results of the multivariable modeling. The use of sleep aids was more likely among respondents who reported work-related emotional stress, family commitments, or changing circadian rhythms to be causes of fatigue or difficulty initiating sleep than among residents who did not. Respondents from programs with a PGY 1–4 format were more likely to use sleep aids than participants in three-year programs. For the use of sleep aids inclusive of alcohol consumption, older respondents were less likely to use sleep aids.

## Discussion

The purpose of this study was to estimate the prevalence of sleep aid use among emergency medicine residents. Our main finding is that among respondents, 46% used sleep aids inclusive of alcohol, while 39% used sleep aids exclusive of alcohol. While reported sleep aids in our study were primarily over-the-counter medications, many prescription medications were used, including oxycodone, codeine, and benzodiazepines. These findings should be tempered by the self-reported nature of sleep aid use. For example, no respondents in our survey reported use of illicit substances, while prior research suggests Emergency Medicine residents are more likely to use cocaine and marijuana than residents from other specialties [19].

Participants whose difficulty initiating sleep was related to changing circadian rhythms were more likely to use sleep aids. Shift work is often cited as one of the most difficult aspects of life as an Emergency Physician, and adverse effects of shift work have long been known [20-23]. Interestingly, fatigue or difficulty initiating sleep due to work-related emotional stress and family commitments were also found to be independent predictors of sleep aid use. While interventions to minimize the impact of circadian rhythm changes on performance are achievable and may aid in improving resident well-being and reducing fatigue-related performance errors [16,24], interventions to reduce the work-related emotional stress and the impact of familial commitments on fatigue and sleep may also be of benefit.

This is the first known study to explore the use of sleep aids among Emergency Medicine residents, or among subject groups known to be at risk of fatigue and sleepiness; there is a dearth of data against which to compare our results. Sleep aid use in EM residents responding to this survey was higher than that reported for a general population survey of adults aged 18–45 in Detroit; in that study 18% used medications, 13% used alcohol, and 5% used medications and alcohol to facilitate sleep [25]. The higher prevalence of sleep aid use in our study may be associated with the stressors of shift work and the emotional stress of the emergency department. While we can formulate hypotheses on cause and effect of sleep aid use, it should be emphasized that our findings are only associative in nature. Further work is needed to explore predictors of sleep aid use, and how sleep aid use may affect performance in resident physicians.

There are several limitations to this study. The primary limitation is the low response rate (16%). The aim of our survey was to estimate the proportion of residents who use sleep aids. The number of residents we found to use sleep aids was 278 inclusive of alcohol. The lowest estimate of the true population proportion can be determined as the ratio of respondents using sleep aids to the entire EM resident population. It might be reasonable to assume, therefore, that at least 7.3% (95 CI 6.5–8.2%) of EM residents were using asleep aids at the time of this survey.

The direction of response bias arising from the low response in this study is not possible to ascertain; in comparison to eligible respondents, we achieved a similar demographic and experience distribution. However, we do not know whether responders were more or less likely to be sleep aid users than non-responders. Factors influencing response bias in this survey include distribution of the survey through Residency Directors. Every attempt was made to contact residents directly but concerns over privacy and local privacy policies prevented us from obtaining more than a partial list of residents' e-mail addresses. It is not possible for us to confirm that Program Directors forwarded the survey URL to their residents. By electing an anonymous survey methodology, it was not possible to ascertain from which programs residents participated. Further, a number of Program Directors did not respond to our initial communication requesting resident e-mail addresses, but instead inappropriately forwarded this request directly to their residents. This likely led to some confusion among residents, and to residents at some programs distrusting the project and its performance. We aimed to maximize the response rate by making the survey anonymous, offering participation in a drawing for a $100 gift certificate, and using regular reminders to prompt response. The sensitive nature of questions and lack of willingness of Emergency Medicine residents to participate in survey research may have further added to the low response.

In addition to response bias, the self-reported nature of the data is like to have led to under reporting of sleep aid use. Prior studies have surveyed drug use in both physicians and nurses [13], and while the use of anonymous surveys has been found to have sensitivity similar to toxicology screens via random urinalysis [26,27], underestimation of use rather than overestimation is the trend found in these studies [28]. Finally, the use of a cross-sectional survey that was designed to estimate prevalence of sleep aid use is a limitation in the analysis of predictors of sleep aid use. Due to the limitations of low response rate, the use of across-sectional survey design, and the likely under reporting of sleep aid use, our results should be interpreted cautiously.

## Conclusion

Even with a low response rate, our data suggest sleep aid use among EM residents may be common. How this affects performance, well-being, and health remains unknown.

## Competing interests

The author(s) declare that they have no competing interests.

## Authors' contributions

DH and CJL conceived and designed the study. DH, AR, and CJL drafted the manuscript. DH and AR conducted the study under the guidance of CJL. DH, AR, and CJL critically reviewed the manuscript. CJL managed the data and conducted statistical analyses. DH takes responsibility for the manuscript as a whole.

## Pre-publication history

The pre-publication history for this paper can be accessed here:



## Supplementary Material

Additional file 1 Appendix A: Survey items. Click here for file

## Figures and Tables

**Table 1:** Causes of fatigue and difficulty initiating sleep, and methods used to initiate sleep among the 602 responding to multiple choice questions.

**Causes of fatigue or difficulty initiating sleep**	**Fatigue during waking hours**	**Difficulty initiating sleep**
Work hours and/or demands of work	84	41
Emotional stress from work related activities	53	49
Family commitments	49	27
Changing circadian rhythms	85	66
Other	4	1
Maintaining sociality	1.3	-
Relationship problems	0.5	-
Eating habits	0.7	-
Financial stress	0.3	0.2
Medications	0.4	-
Hormonal cycles	0.2	-
Stress over household maintenance	-	0.3
Lack of exercise	-	0.2
Pregnancy	-	0.2
Health problems	-	0.2

All numbers are percentages; respondents could provide more than one answer for each question.

**Table 2:** Responses to detailed questions on use of sleep aids and responses to the direct questions asking whether or not medications or alcohol were used to help initiate sleep.

		**Sleep aid use based on detailed questions (Questions 9 to 11)**			
		
		**No**	**Yes**	**No response**	**Total**
**Use of alcohol or medication to help initiate sleep (Question 7)**	**No**	182	***67***	134	383
	**Yes**	***16***	***184***	***7***	207
	**No response**	5	***4***	3	12
	**Total**	203	255	144	602

The numbers in ***bold italics ***represent the maximum number of all respondents that may be considered to use a sleep aid (N = 278).

**Table 3:** Univariable logistic regression showing the influence of year of training, program type, moonlighting, causes of fatigue, causes of difficulty initiating sleep, and demographics on the odds of using sleep aids either inclusive or exclusive of alcohol.

		**Odds Ratio**	**95% confidence interval**
		
**Inclusive of alcohol**			
Year of residency	2nd year v 1st year	0.95	(0.64 – 1.43)
	3rd year v 1st year	0.99	(0.66 – 1.50)
	4th year v 1st year	1.48	(0.77 – 2.85)
Type of program	PGY1–4 v PGY1–3	1.72	(1.07 – 2.74)
	PGY2–4 v PGY1–3	1.23	(0.77 – 1.97)
Moonlighting	Yes v no	0.88	(0.57 – 1.38)
Causes of fatigue	Work hours/demands of work	1.93	(1.22 – 3.05)
	Work-related emotional stress	2.10	(1.51 – 2.92)
	Family commitments	1.48	(1.07 – 2.05)
	Changing circadian rhythms	2.14	(1.32 – 3.47)
Causes of difficulty initiating sleep	Work hours/demands of work	2.29	(1.64 – 3.21)
	Work-related emotional stress	2.14	(1.54 – 2.98)
	Family commitments	1.72	(1.19 – 2.49)
	Changing circadian rhythms	2.61	(1.83 – 3.73)
Sleepiness	ESS 11–16 v ESS 0–10	0.77	(0.55 – 1.08)
	ESS > 16 v ESS 0–10	0.91	(0.48 – 1.72)
Demographics	Age	0.97	(0.93 – 1.01)
	Female v Male	1.13	(0.81 – 1.57)
	White v Black	1.25	(0.50 – 3.11)
	Asian v Black	1.16	(0.52 – 2.59)
	Other v Black	1.07	(0.35 – 3.25)
	Non-Hispanic v Hispanic	1.56	(0.65 – 3.76)
**Exclusive of alcohol**			
Year of residency	2nd year v 1st year	1.03	(0.68 – 1.55)
	3rd year v 1st year	1.16	(0.76 – 1.77)
	4th year v 1st year	1.50	(0.78 – 2.89)
Type of program	PGY1–4 v PGY1–3	1.79	(1.12 – 2.86)
	PGY2–4 v PGY1–3	1.35	(0.84 – 2.16)
Moonlighting	Yes v no	0.87	(0.55 – 1.37)
Causes of fatigue	Work hours/demands of work	1.79	(1.11 – 2.89)
	Work-related emotional stress	1.88	(1.34 – 2.64)
	Family commitments	1.50	(1.08 – 2.10)
	Changing circadian rhythms	1.82	(1.10 – 3.00)
Causes of difficulty initiating sleep	Work hours/demands of work	2.10	(1.50 – 2.95)
	Work-related emotional stress	1.88	(1.34 – 2.62)
	Family commitments	1.72	(1.19 – 2.49)
	Changing circadian rhythms	2.43	(1.68 – 3.53)
Sleepiness	ESS 11–16 ESS 0–10	0.80	(0.56 – 1.13)
	ESS > 16 ESS 0–10	0.94	(0.49 – 1.80)
Demographics	Age	0.97	(0.93 – 1.02)
	Female v Male	1.38	(0.98 – 1.93)
	White v Black	2.70	(0.96 – 7.58)
	Asian v Black	2.09	(0.82 – 5.31)
	Other v Black	2.22	(0.66 – 7.48)
	Non-Hispanic v Hispanic	1.42	(0.57 – 3.51)

**Table 4:** Multivariable logistic regression models for predicting the odds of using sleep aids, either inclusive or exclusive of alcohol.

		**Sleep aids inclusive of alcohol**		**Sleep aids exclusive of alcohol**	
		
		**Odds Ratio**	**95% confidence interval**	**Odds Ratio**	**95% confidence interval**
Type of program	PGY1–4 v PGY1–3	2.09	(1.17 – 3.72)	1.82	(1.03 – 3.22)
	PGY2–4 v PGY1–3	1.43	(0.81 – 2.53)	1.31	(0.75 – 2.30)
Causes of fatigue	Work-related emotional stress	2.03	(1.35 – 3.04)	1.74	(1.15 – 2.64)
	Family commitments			1.41	(0.94 – 2.11)
Causes of difficulty initiating sleep	Family commitments	1.76	(1.13 – 2.73)		
	Changing circadian rhythms	2.05	(1.33 – 3.16)	1.99	(1.28 – 3.08)
Age		0.95	(0.89 – 1.00)		

## References

[B1] Veasey S, Rosen R, Barzansky B, Rosen I, Owens J (2002). Sleep loss and fatigue in residency training: a reappraisal. JAMA.

[B2] Lockley SW, Cronin JW, Evans EE, Cade BE, Lee CJ, Landrigan CP, Rothschild JM, Katz JT, Lilly CM, Stone PH, Aeschbach D, Czeisler CA, Harvard Work Hours, Health and Safety Group (2004). Effect of reducing interns' weekly work hours on sleep and attentional failures. N Engl J Med.

[B3] Landrigan CP, Rothschild JM, Cronin JW, Kaushal R, Burdick E, Katz JT, Lilly CM, Stone PH, Lockley SW, Bates DW, Czeisler CA (2004). Effect of reducing interns' work hours on serious medical errors in intensive care units. N Engl J Med.

[B4] Defoe DM, Power ML, Holzman GB, Carpentieri A, Schulkin J (2001). Long hours and little sleep: work schedules of residents in obstetrics and gynecology. Obstet Gynecol.

[B5] Godellas CV, Huang R (2001). Factors affecting performance on the American Board of Surgery in-training examination. Am J Surg.

[B6] Cavallo A, Jaskiewicz J, Ris MD (2002). Impact of night-float rotation on sleep, mood, and alertness: the resident's perception. Chronobiol Int.

[B7] Howard SK, Gaba DM, Rosekind MR, Zarcone VP (2002). The risks and implications of excessive daytime sleepiness in resident physicians. Acad Med.

[B8] Owens JA (2001). Sleep loss and fatigue in medical training. Curr Opin Pulm Med.

[B9] Barger LK, Cade BE, Ayas NT, Cronin JW, Rosner B, Speizer FE, Czeisler CA, Harvard Work Hours, Health, and Safety Group (2005). Extended Work Shifts and the Risk of Motor Vehicle Crashes among Interns. N Engl J Med.

[B10] Steele MT, Ma OJ, Watson WA, Thomas HA, Muelleman RL (1999). The occupational risk of motor vehicle collisions for emergency medicine residents. Acad Emerg Med.

[B11] Frese M, Okonek KA (1984). Reasons to leave shiftwork and psychological and psychosomatic complaints of former shiftworkers. J Appl Psychol.

[B12] Kawachi I, Colditz GA, Stampfer MJ, Willett WC, Manson JE, Speizer FE, Hennekens CH (1995). Prospective study of shift work and risk of coronary heart disease in women. Circulation.

[B13] Trinkoff AM, Storr CL (1997). Collecting substance use data with an anonymous mailed survey. Drug Alcohol Depend.

[B14] Trinkoff AM, Storr CL (1998). Work schedule characteristics and substance use in nurses. Am J Ind Med.

[B15] Gordon N, Cleary P, Parker C, Czeisler CA (1986). The prevalence and health inpact of shift work. Am J Public Health.

[B16] Arora V, Dunphy C, Chang VY, Ahmad F, Humphrey HJ, Meltzer D (2006). The effects of on-duty napping on intern sleep time and fatigue. Ann Intern Med.

[B17] Johns MW (1991). A new method for measuring daytime sleepiness: the Epworth Sleepiness Scale. Sleep.

[B18] (2004). Accreditation Council for Graduate Medical Education (ACGME) Accreditation Data System, Chicago.

[B19] Hughes PH, Baldwin DC, Sheehan DV, Conard S, Storr CL (1992). Resident physician substance use, by specialty. Am J Psychiatry.

[B20] Steele MT, Ma OJ, Watson WA, Thomas HA (2000). Emergency medicine residents' shiftwork tolerance and preference. Acad Emerg Med.

[B21] Costa G (1997). The problem: shiftwork. Chronobiol Int.

[B22] Tepas DI, Carvalhais AB (1990). Sleep patterns of shiftworkers. Occup Med.

[B23] Monk TH (1990). Shiftworker performance. Occup Med.

[B24] Smith-Coggins R, Howard SK, Kwan S, Wang C, Mac DT, Rosekind MR, Sowb YA, Balise R, Gaba DM (2002). Do Naps during the Night Shift Improve Performance in the Emergency Department?. Acad Emerg Med.

[B25] Johnson EO, Roehrs T, Roth T, Breslau N (1998). Epidemiology of alcohol and medication as aids to sleep in early adulthood. Sleep.

[B26] Lehman WEK, Simpson DD (1992). Employee substance use and on-the-job behaviors. J Appl Psychol.

[B27] Cook RF, Bernstein AD, Arrington TL, Andrews CM, Marshall GA (1995). Methods for assessing drug use prevalence in the workplace: A comparison of self-report, urinalysis, and hair analysis. Int J Addict.

[B28] Turner CF, Lessler JT, Gfroerer J "Survey Measurement of Drug Use: Methodological Studies.". DHHS Publ (ADM) 92-1929.

