# Supplementary material for: The use of sleep aids among Emergency Medicine residents: a web based survey
Source: BMC Health Serv Res. 2006 Oct 19;6:136. doi: 10.1186/1472-6963-6-136 (PMC1657010; doi:10.1186/1472-6963-6-136)
Supplement: Additional file 1 — Appendix A: Survey items. [file 1472-6963-6-136-S1.doc]

**Appendix A: Survey Items**

1) What year of residency are you in: 🖵 PGY-1 🖵 PGY-2 🖵 PGY-3 🖵 PGY-4

2) What kind of program do you attend: 🖵 PGY1-3 🖵 PGY1-4 🖵 PGY2-4

3) Do you participate in additional ED shifts beyond those

required by your residency program? 🖵 Yes 🖵 No

The following questions apply to your experiences **with ED months during your residency**.

4) How long, on average, does it take you to fall asleep? *(Check One)*

| >10 minutes | 🖵 |
| --- | --- |
| 5-10 minutes | 🖵 |
| <5 minutes | 🖵 |

| 5) | Do any of the following cause you **fatigue during waking hours**? Answer yes if this occurs more than 4 times a month for at least two consecutive months. *(Answer yes or no for every question)* | | | |
| --- | --- | --- | --- | --- |
|  |  | | **Yes** | **No** |
|  | a) | Work hours and/or demands of work | 🖵 | 🖵 |
|  | b) | Emotional stress from work-related activities | 🖵 | 🖵 |
|  | c) | Family commitments | 🖵 | 🖵 |
|  | d) | Changing circadian rhythms | 🖵 | 🖵 |
|  | e) | Other (please specify) _______________________________ | 🖵 | 🖵 |
|  |  |  |  |  |
|  |  |  |  |  |
| 6) | Do any of the following cause you **difficulty with initiating sleep**? Answer yes if this occurs more than 4 times a month for at least two consecutive months. *(Answer yes or no for every question)* | | | |
|  |  | | **Yes** | **No** |
|  | a) | Work hours and/or demands of work | 🖵 | 🖵 |
|  | b) | Emotional stress from work-related activities | 🖵 | 🖵 |
|  | c) | Family commitments | 🖵 | 🖵 |
|  | d) | Changing circadian rhythms | 🖵 | 🖵 |
|  | e) | Other (please specify) _______________________________ | 🖵 | 🖵 |

| 7) | Do you use any of the following methods regularly to **help you fall asleep**? Answer yes if you used the method more than 4 times a month for at least two consecutive months. *(Answer yes or no for every question)* | | | |
| --- | --- | --- | --- | --- |
|  |  | | **Yes** | **No** |
|  | a) | Watching television | 🖵 | 🖵 |
|  | b) | Reading | 🖵 | 🖵 |
|  | c) | Meditation, yoga, or some other sort of exercise | 🖵 | 🖵 |
|  | d) | Sexual activity | 🖵 | 🖵 |
|  | e) | Medication (over the counter or prescription) | 🖵 | 🖵 |
|  | f) | Alcohol use | 🖵 | 🖵 |
|  | g) | Other (please specify) _______________________________ | 🖵 | 🖵 |

| 8) | Using the scale provided, how likely are you to **doze off or fall asleep** in the following situations, in contrast to feeling just tired? This refers to your usual way of life. Even if you can’t remember doing some of these things, try to work out how they would affect you. *(Write the number corresponding to your choice in the space provided, answer all questions)* | | |
| --- | --- | --- | --- |
|  | | **0** | **1** | **2** | **3** | | --- | --- | --- | --- | | **no** chance of sleeping | **slight** chance of sleeping | **moderate** chance of sleeping | **high** chance of sleeping | | | |
|  |  | | **Chance of Sleeping** |
|  | a) | Sitting and reading | ___ |
|  | b) | Watching TV | ___ |
|  | c) | Sitting inactive in a public place (e.g a theater or a meeting) | ___ |
|  | d) | As a passenger in a car for an hour without a break | ___ |
|  | e) | Lying down to rest in the afternoon when circumstances permit | ___ |
|  | f) | Sitting and talking to someone | ___ |
|  | g) | Sitting quietly after a lunch without alcohol | ___ |
|  | h) | In a car, while stopped for a few minutes in traffic | ___ |
|  |  |  |  |

The following questions ask about any over the counter medications, prescription medications, and non-pharmaceutical substances you use to help you fall asleep, stay asleep or stay alert. We are interested only about the time since you began residency. All answers are anonymous

| 9) | Do you **ever** use **over the counter medications** to help you fall asleep, stay asleep or stay alert?  *(OTC drugs include Benadryl, Tylenol PM etc…)* | | | | | | | | | **Yes** 🖵 | | **No** 🖵 | |  |
| --- | --- | --- | --- | --- | --- | --- | --- | --- | --- | --- | --- | --- | --- | --- |
|  | | If ***yes***, give the following information for each over the counter medication you used | | | | | | | | | | | |  |
|  | | Name of Substance | | | Number of times used **per month** | | Used to fall asleep | | Used to stay asleep | | | Used to stay alert | |  |
|  | |  | | | 🖵 < 5  🖵 5-10  🖵 > 10 | | 🖵 | | 🖵 | | | 🖵 | |  |
|  | |  | | | 🖵 < 5  🖵 5-10  🖵 > 10 | | 🖵 | | 🖵 | | | 🖵 | |  |
|  | |  | | | 🖵 < 5  🖵 5-10  🖵 > 10 | | 🖵 | | 🖵 | | | 🖵 | |  |
|  | |  | | | 🖵 < 5  🖵 5-10  🖵 > 10 | | 🖵 | | 🖵 | | | 🖵 | |  |
|  | |  | | | 🖵 < 5  🖵 5-10  🖵 > 10 | | 🖵 | | 🖵 | | | 🖵 | |  |
| 10) | Do you **ever** use **prescription medications** to help you fall asleep, stay asleep or stay alert?  *(Prescription medications include Ambien, Valium etc…)* | | | | | | | | | **Yes** 🖵 | | **No** 🖵 | | |
|  | | | If ***yes***, give the following information for each prescription medication you used | | | | | | | | | |  | |
|  | | | Name of Substance | Number of times used **per month** | | Used to fall asleep | | Used to stay asleep | | | Used to stay alert | |  | |
|  | | |  | 🖵 < 5  🖵 5-10  🖵 > 10 | | 🖵 | | 🖵 | | | 🖵 | |  | |
|  | | |  | 🖵 < 5  🖵 5-10  🖵 > 10 | | 🖵 | | 🖵 | | | 🖵 | |  | |
|  | | |  | 🖵 < 5  🖵 5-10  🖵 > 10 | | 🖵 | | 🖵 | | | 🖵 | |  | |
|  | | |  | 🖵 < 5  🖵 5-10  🖵 > 10 | | 🖵 | | 🖵 | | | 🖵 | |  | |
|  | | |  | 🖵 < 5  🖵 5-10  🖵 > 10 | | 🖵 | | 🖵 | | | 🖵 | |  | |

| 11) | Do you **ever** use **non-pharmaceutical substances** to help you fall asleep, stay asleep or stay alert?  *(non-pharmaceutical substances include alcohol, tobacco, caffeine etc…)* | | | | | **Yes** 🖵 | | **No** 🖵 |
| --- | --- | --- | --- | --- | --- | --- | --- | --- |
|  | | If ***yes***, give the following information for each non-pharmaceutical substance you used | | | | | | |
|  | | Name of Substance | Number of times used **per month** | Used to fall asleep | Used to stay asleep | | Used to stay alert | |
|  | |  | 🖵 < 5  🖵 5-10  🖵 > 10 | 🖵 | 🖵 | | 🖵 | |
|  | |  | 🖵 < 5  🖵 5-10  🖵 > 10 | 🖵 | 🖵 | | 🖵 | |
|  | |  | 🖵 < 5  🖵 5-10  🖵 > 10 | 🖵 | 🖵 | | 🖵 | |
|  | |  | 🖵 < 5  🖵 5-10  🖵 > 10 | 🖵 | 🖵 | | 🖵 | |
|  | |  | 🖵 < 5  🖵 5-10  🖵 > 10 | 🖵 | 🖵 | | 🖵 | |

**Demographic Information:**

**Age: __ __ years**

| Gender: | **Male** | **Female** |
| --- | --- | --- |
| *(check one)* | 🖵 | 🖵 |

| Race: | **African American** | **Asian** | **Caucasian** | **Native American** | **Other** |
| --- | --- | --- | --- | --- | --- |
| *(check all that apply)* | 🖵 | 🖵 | 🖵 | 🖵 | 🖵 |

| Ethnicity: | **Hispanic** | **Not Hispanic** |
| --- | --- | --- |
| *(check one)* | 🖵 | 🖵 |
